# Formation of High-Conductive C Subunit Channels upon Interaction with Cyclophilin D

**DOI:** 10.3390/ijms222011022

**Published:** 2021-10-13

**Authors:** Giuseppe Federico Amodeo, Natalya Krilyuk, Evgeny V. Pavlov

**Affiliations:** Department of Molecular Pathobiology, New York University, New York, NY 10012, USA; natalya.krilyuk@gmail.com (N.K.); ep37@nyu.edu (E.V.P.)

**Keywords:** amyloids, permeability transition pore, mitochondrial dysfunction, misfolded proteins

## Abstract

The c subunit of the ATP synthase is an inner mitochondrial membrane (IMM) protein. Besides its role as the main component of the rotor of the ATP synthase, c subunit from mammalian mitochondria exhibits ion channel activity. In particular, c subunit may be involved in one of the pathways leading to the formation of the permeability transition pore (PTP) during mitochondrial permeability transition (PT), a phenomenon consisting of the permeabilization of the IMM due to high levels of calcium. Our previous study on the synthetic c subunit showed that high concentrations of calcium induce misfolding into cross-β oligomers that form low-conductance channels in model lipid bilayers of about 400 pS. Here, we studied the effect of cyclophilin D (CypD), a mitochondrial chaperone and major regulator of PTP, on the electrophysiological activity of the c subunit to evaluate its role in the functional properties of c subunit. Our study shows that in presence of CypD, c subunit exhibits a larger conductance, up to 4 nS, that could be related to its potential role in mitochondrial toxicity. Further, our results suggest that CypD is necessary for the formation of c subunit induced PTP but may not be an integral part of the pore.

## 1. Introduction

Mitochondrial stress induced by high concentrations of calcium and/or reactive oxygen species leads to the permeabilization of the inner mitochondrial membrane (IMM), a phenomenon called permeability transition (PT) [1,2]. PT occurs through the opening of a large channel, namely the permeability transition pore (PTP), across the IMM that allows the free flow of small metabolites (up to 1.5 kDa) and ions causing the dissipation of the membrane potential and disruption of oxidative phosphorylation [3]. The molecular organization of the PTP is not completely understood. It has been proposed that PTP can be formed by different proteins such as the adenosine nucleotide translocase (ANT), monomers or dimers of the ATP synthase and sole c subunit of the ATP synthase [1,4,5,6,7]. Other studies suggest a separate mechanism where amyloidogenic peptides are involved in the formation of PTP in aging-related disorders. Interestingly, we discovered that the c subunit of the ATP synthase is in fact an amyloidogenic peptide [8,9]. Despite PTP ion conducting pores formed by these protein complexes exhibit different conductances, their involvement in the channel opening depends on the interaction with cyclophilin D (CypD), a mitochondrial chaperone and the specific target of cyclosporin A (CSA) [10]. We recently showed that the unmodified c subunit is an amyloidogenic ion-channel forming peptide whose oligomers exhibited conductance of up to 500 pS. Here, we aim to establish the effect of CypD on the activity of the c subunit in model lipid bilayers. The incubation of the c subunit with CypD led to the formation of a much larger channel with conductances up to 4 nS. However, the addition of CSA did not affect the channel activity, suggesting that CypD may be essential for the formation of PTP but is not a structural component of the pore itself.

## 2. Results

### 2.1. CypD Induces the Formation of High-Conductive C Subunit

The c subunit of the ATP synthase is a 75 amino acids short peptide that, in native conditions, folds into an α-helical hairpin and assembles into oligomers, the so-called c-rings, which is the major component of the F_0_ complex [11]. Its physiological role is to allow the transport of protons into the matrix in the process coupled to ATP production by ATP synthase. In addition to its role in the oxidative phosphorylation, several independent studies reported that c subunit may be involved in the formation of PTP under pathological conditions [6,12,13,14]. One of the mechanisms of c subunit participation is through opening of an ion conductive pore within the native c-ring of the ATP synthase. This mechanism requires disassembly of the complete ATPase and opening of the pore [4,15]. An alternative mechanism is the direct formation of the pore by oligomerization of the c subunit monomers. This mechanism is supported by studies that showed that the c subunit alone is sufficient to induce PTP [6,12,14]. Our recent report showed that the unmodified c subunit is an amyloidogenic peptide that spontaneously folds into cross-β oligomers and offers a potential mechanism on how such pores can form [8]. One of the defining features of the channel candidate for involvement in PTP is its dependence on CypD, an endogenous mitochondrial chaperone and well-established activator of PTP [16]. Administration of CSA, an inhibitor of CypD, in cells overexpressing the c subunit resulted in a more effective prevention of PTP opening, suggesting a direct interaction between the protein and the chaperone [12]. Thus, in order to explore the possible link between c subunit and PT, we tested here how the channel activity of c subunit oligomers is modulated by CypD.

**Figure 1:**
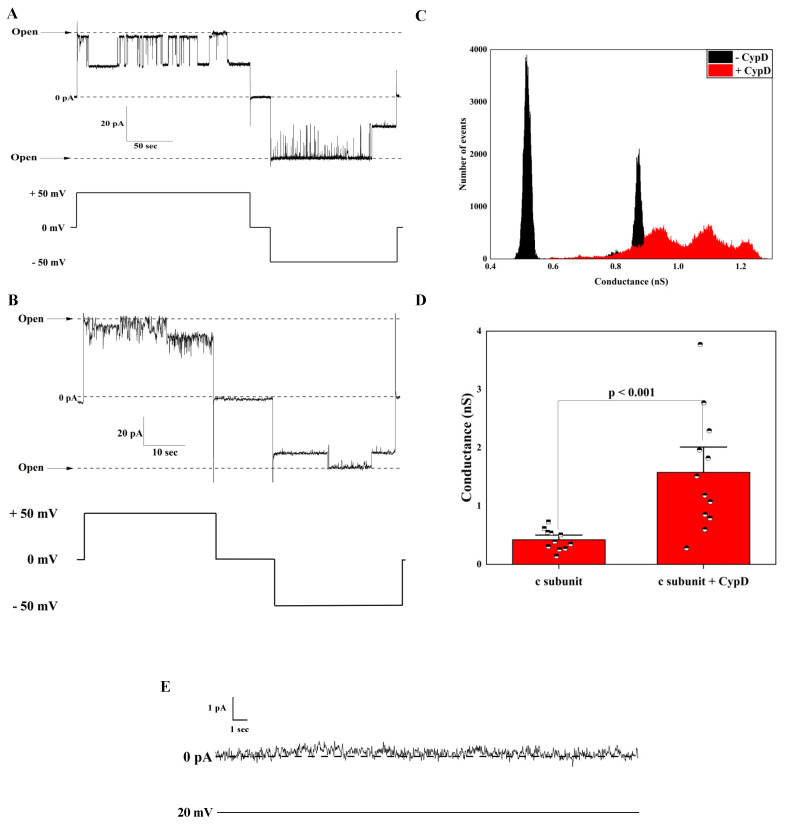
Modulation of the c subunit ion channel activity upon interaction with CypD. (**A**) Representative current trace of oligomers showing typical channel behavior with frequent transitions between fully open and lower conductance states; (**B**) Representative current trace of c subunit oligomers preincubated with CypD; (**C**) All points histogram corresponding to the traces shown in panel A and B; (**D**) Channel open state conductance values of the c subunit oligomers alone (*n* = 11) and in presence of CypD (*n* = 12); (**E**) Control trace of the bilayer prior to the channel insertion.

**Figure 2:**
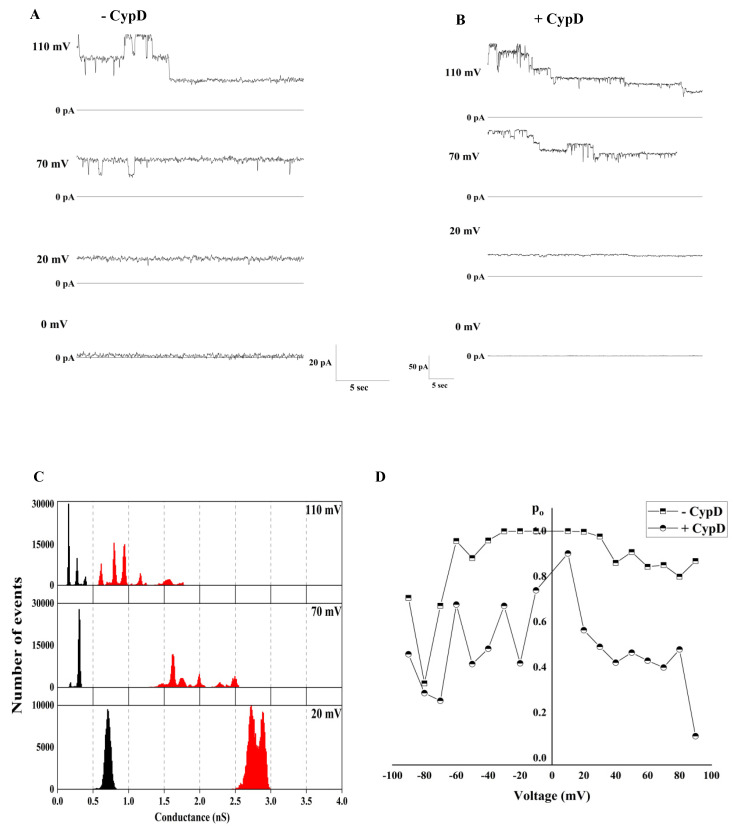
Voltage-dependence of c subunit channels before and after preincubation with CypD. (**A**) Representative c subunit oligomers activity at different voltages before and (**B**) after preincubation with CypD; (**C**) all points histogram corresponding to the traces shown in panel A and B; (**D**) voltage dependence of the open probability of the c subunit channel.

**Figure 3:**
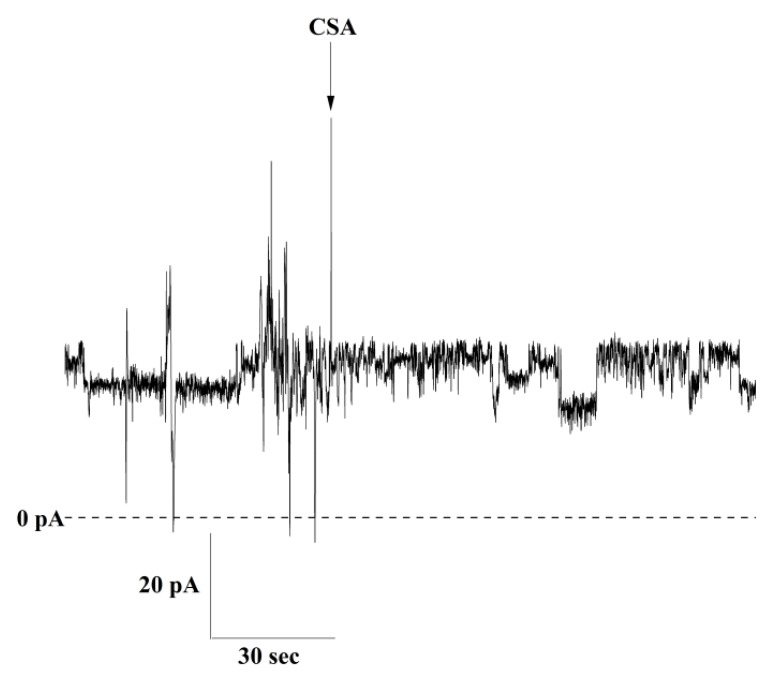
Effect of PTP inhibitor CSA. Ion current trace of the c subunit preincubated with CypD showing channel activity before and after addition of CSA (20 µM).

To test the effects of CypD, we first recorded the activity of the channels formed by c subunit in the absence of the chaperone. To do this, we refolded the unmodified synthetic c subunit by resuspending the lyophilized peptide in a buffer containing 0.14% n-dodecyl β-D-maltoside (DDM) in PBS to mimic the membrane environment and 1 mM Ca^2+^ to inhibit fibrillation. Upon reconstitution into artificial lipid bilayers, we observed channels with an average conductance ranging from 300 pS up to 800 pS with an average conductance of 400 pS (*n* = 11). Figure 1A shows a representative trace of the c subunit oligomers at 50 mV. This channel activity is in good agreement with previously reported activity of the unmodified peptide [8]. In order to study the effect of CypD on the electrophysiological activity of the c subunit, we dissolved the lyophilized peptide in the same buffer as above, but this time, it was supplemented with 0.41 mg/mL of CypD. Figure 1B shows a representative trace of the channel activity in the presence of CypD. When compared to the control c subunit, these channels had a higher average conductance of about 1.5 nS (*n* = 12) and wider distribution of the conductances ranging from 400 pS up to about 4 nS. We suggest the possibility that this increase in the range of conductances in the presence of CypD is due to the formation of higher order oligomers. However, testing this hypothesis would require further investigation. Both preparations showed a similar voltage dependency with the channels switching to lower conductance substates at high voltages (Figure 2A–D). However, the V_h-i_ decreased from −75 ± 1 mV to −46 ± 9 mV in the presence of CypD, indicating that a higher number of channels were closed at lower voltages (*n* = 5). These results suggest that CypD may induce the formation of higher order oligomers with different stoichiometry of the c subunit with a PTP-like activity. Next, we used CSA in order to gain insights on the role of CypD in the channel formation/activity. In our experiments, we added CSA directly to the recording solution to the final concentration of 20 µM. Over independent experiments (*n* = 10), we observed that CSA had no effect on the channel activity except in one case (Figure 3). This result supports the idea that CypD, which is the specific target of CSA, is not required for channel activity but likely modifies the pore assembly during c subunit oligomerization. The non-essential role of CypD in channel activity is consistent with previous studies that showed that the permeabilization of the IMM can occur even in the absence of CypD through the opening of channels with a lower conductance compared to the one of PTP. However, we should note that the lack of CSA effect on the channel activity does not completely exclude the possibility of CypD as a structural component of the complex. Importantly, preincubation of the c subunit with both CSA and CypD resulted in no channel activity, suggesting that CSA affects CypD-c subunit interactions during preincubation, presumably at the channel formation stage. Based on these results, further studies to determine the specific protein-protein interactions between the c subunit and CypD are required, particularly considering that the primary sequence of the c subunit carries only one proline, the substrate of the chaperone, close to the loop of the native α-helical hairpin conformation. The single mutation at that position may provide useful insights on the nature of the interaction between the two proteins.

### 2.2. Role of Misfolded Proteins in PT

The idea that PT can occur by different molecular pathways has gained more experimental evidence in recent years [13,15,17,18,19]. It has been demonstrated that CypD dependent PTP can be formed by ANT and ATPase transformation into large pores [4,7,18,19,20,21,22]. Our study suggests that CypD dependent channel activity resembling PTP can also be formed by the misfolded c subunit. In this scenario, due to its amyloidogenic properties, the c subunit can form channels via an independent pathway, and this process is modulated by the interaction with CypD.

The possibility of misfolded proteins being responsible for PTP formation has been proposed before by He and Lemasters [23]. In their study, they report that high concentrations of reactive oxygen species lead to the misfolding of native mitochondrial membrane proteins (e.g., ANT, VDAC, and maybe other proteins) that in turn aggregate into clusters in the IMM. Such clusters can undergo into two different pathways: the formation of either unregulated or regulated PT pores. The opening of an unregulated pore occurs spontaneously while the formation of a regulated PTP occurs upon interaction with mitochondrial chaperones. In particular, CypD and other chaperones bind to these misfolded clusters to refold them into their native conformation. Finally, the binding of Ca^2+^ to CypD induces the opening of PTP [23].

As one of the most abundant amyloidogenic protein in the inner membrane, the c subunit might be the principal peptide involved in PTP misfolded protein pathway [24]. However, there is clear evidence in literature that a similar mechanism can involve other amyloidogenic peptides [9]. Indeed, an increasing number of studies on neurodegenerative diseases show that PT plays a significant role in cell death. In Alzheimer’s disease models, amyloid β induces high concentrations of ROS and Ca^2+^ in the mitochondrial matrix and binds to CypD to form a complex that leads to the opening of PTP [25]. Similar results were obtained with Parkinson’s disease models showing that misfolded α-synuclein oligomers formation was Ca^2+^-dependent and induced mitochondrial cell death by activating PTP [26,27]. Specifically, toxic oligomeric forms of α-synuclein activate PTP in the intact mitochondria and cause CSA dependent channel formation when added directly to the patch pipette containing mitochondrial inner membranes. The unmodified c subunit of the ATPase has the same amyloidogenic properties as both amyloid β and α-synuclein, including fibril and oligomer formation and permeabilization of lipid membranes. This result, in combination with the previous findings on amyloidogenic peptides, endorses the notion that, in addition to ANT and ATPase pathways, PT may be caused by protein misfolding and their interaction with the mitochondrial chaperone CypD [21]. In fact, it has been shown that CSA is able to inhibit the pore opening at an early stage but not at later ones, suggesting that it can inhibit the folding activity of CypD [28]. Our results support the idea that CypD is not an integral part of the pore formed by the misfolded c subunit but plays an essential role in folding the high-conductive unregulated PTP. Finally, it is worth noting that recent estimates suggest that each mitochondrion has a very low number of PTP channels [29]. This observation is consistent with the possibility that most of the mitochondrial copies of the c subunit are present in fully folded form and, thus, cannot be easily converted into misfolded conformation, which can be achieved only by a very small subpopulation of peptides that are not present in physiological form.

## 3. Materials and Methods

### 3.1. Chemicals

Synthetic peptides with free termini were purchased from LifeTein Inc. (Somerset, NJ, USA) with a purity of 98%. Recombinant Cyclophilin D, expressed in *E. coli*, was purchased at Sigma (SRP6067) with a purity ≥95%. All other chemicals were purchased at Sigma.

### 3.2. BLM Recordings

The painting method was used to form phospholipid bilayer using 1,2-diphytanoyl-*sn*- glycero-3-phosphocholine (DiPhPC, Avanti Polar Lipids). The bilayer was formed at the 50–100 µm diameters apertures of Delrin cuvettes as previously described [30]. Briefly, the aperture was pre-treated with 25 mg/mL of DiPhPC in decane and allowed to dry. Bilayers were formed using the painting method after filling up the cuvettes with the recording solution (150 mM KCl, 20 mM HEPES, pH 7.4) on both sides of the chamber. Protein insertions were attained by dipping the protein solution onto the lipid bilayer. Ion currents were measured using standard silver-silver chloride electrodes from WPI (World Precision Instruments) that were placed in each side of the cuvette. Measurements of the conductance of single channels were performed by painting the protein to the cis side of the chamber (the side connected to the ground electrode). Spontaneous channel insertion was typically obtained under an applied voltage of 20 mV. Conductance measurements were performed using an eONE amplifier (Elements) with a sampling rate of 10 kHz (809.1 µs interval). Traces were filtered by a low-pass Bessel filter at 10 Hz for analyses performed with Origin Pro 8 (OriginLab) and Clampfit software (Molecular devices). The steady-state inactivation curves were fitted by the equation: *p_o_* = 1/(1 + exp((*V*_h-I_ − *V*)/*b*)), where *V*_h-I_ is the potential of half-maximal inactivation and *b* is a slope factor (mV/*e*-fold) [31].

## 4. Conclusions

In conclusion, we find that the channel forming activity of the c subunit is modulated by CypD foldase. Specifically, incubation with CypD leads to the formation of pores with significantly larger conductances. These results provide additional evidence on how amyloid peptides might be directly involved in the formation of PTP.

## Data Availability

Not applicable.
